# Impact of the Ripple Effect on the Resilience of Multimodal Container Port Operations: A System Dynamics Simulation Approach

**DOI:** 10.1111/risa.70149

**Published:** 2025-11-21

**Authors:** Jinglin Zhang, Xuri Xin, Rameshwar Dubey, Trung Thanh Nguyen, Xiaoning Shi, Na Li, Zaili Yang

**Affiliations:** ^1^ Liverpool Logistics, Offshore and Marine (LOOM) Research Institute Liverpool John Moores University Liverpool UK; ^2^ Liverpool Business School Liverpool John Moores University Liverpool UK; ^3^ Montpellier Business School Montpellier France; ^4^ Institute of Transport Research German Aerospace Center (DLR) Berlin Germany; ^5^ College of Transportation Engineering Dalian Maritime University Dalian China

**Keywords:** Evidential Reasoning, multimodal container port, resilience assessment, ripple effect, system dynamics

## Abstract

Current assessments of port resilience primarily focus on the risks affecting its operations, often neglecting the ripple effects across different subsystems within a port. In multimodal container ports, these sub‐systems include liner shipping, feeder shipping, railways, and trucking. Moreover, prevailing research predominantly addresses port resilience from a macro perspective without detailing micro‐level operational concerns. This article proposes a new integrated methodology that not only considers but also quantifies the ripple effects across different multimodal sub‐systems and their impact on overall port resilience. It employs real operational and accident data to assess the resilience of a multimodal container port under different disruption scenarios, hence providing valuable insights into preventing systemic failures through targeted interventions at the subsystem level. The proposed methodology comprises three principal components: a system dynamics (SD) simulation that integrates variables and factors affecting port resilience, a resilience analysis model that converts system performance into a resilience metric based on three fundamental criteria, and a comprehensive port system resilience assessment utilizing Evidential Reasoning (ER). Each step, from the detailed simulation model reflecting micro‐level mechanisms to aggregating information across subsystems, builds toward determining the port's overall resilience. Multiple disruptive scenarios are designed and derived from historical failures and field investigations to validate the effectiveness of the proposed methodology. The results demonstrate that the proposed approach effectively assesses port performance under disruptions, identifies critical subsystems, and supports timely recovery strategies. Applicable to other port systems, this approach offers essential insights for improving long‐term resilience in container port operations.

## 1 Introduction

Seaports, critical hubs that link various transportation modes within maritime supply chains, are pivotal to the operational efficiency of the overall supply chain, international trade, and economic growth. Yet, due to their unique geographical locations and geopolitical contexts, port operations often embody high uncertainty (Jiang et al., 2021). The collaborations necessary for loading and unloading operations among various vehicles within ports introduce the risks of transferring the disruptions and impacts that propagate across different transportation modes, a phenomenon referred to as the “ripple effect” (Verschuur et al., 2022). Previous research has identified that various risks, including climate change, labor strikes, terrorism, global pandemics (Zhou et al., 2024; Tan et al., 2022; Yang et al., 2025), and political conflicts (Liu et al., 2025), disrupt port operations (Choi, 2021). Moreover, studies have shown that indirect losses from the ripple effect often exceed direct losses (Afenyo et al., 2022). It demonstrates the urgency for new studies to generate effective strategies designed to anticipate and withstand unforeseen disruptions, absorb their impacts, sustain essential functions, and swiftly recuperate, thereby enhancing port resilience (Cheng et al., 2022).

Foundational studies define port resilience as a combination of the inherent stability and the capacity to implement rapid mitigation strategies (Nair et al., 2010). Over time, this concept has evolved within the transportation field, now offering a comprehensive system‐wide assessment of a system's reliability, vulnerability, and recovery capacity in the face of disruptions. Extensive studies have explored port resilience from qualitative (incl. conceptual), and quantitative perspectives, providing insights from various aspects, including the port‐hinterland container transport network (Chen et al., 2018), capacity sharing, and cross‐port investments during disruptions (Li, Asadabadi, et al., 2022), economic and social impacts of port disruptions (Wei et al., 2022), the Ports Resilience Index (PRI) against climate change in seaports (León‐Mateos et al., 2021), and the prioritization of initiatives based on multi‐scenarios and multi‐stakeholders perspectives (Almutairi et al., 2019). Compared to other metrics that reflect a system's capability to respond to unexpected disruptions (Zhang et al., 2023), resilience captures a broader range of operational attributes and port characteristics. Enhancing resilience first requires advancing assessment capabilities through practical and efficient evaluation methods (Wang and Yuen, 2022; Nosrati Malekjahan et al., 2025). Accordingly, this research endeavors to propose a novel assessment methodology for port resilience, which, to the best of the authors’ understanding, is the first to address how ripple effects propagate among multiple transportation modes within ports, thereby facilitating the development of response and recovery strategies.

Despite significant interest from both industry and academia in managing port risks and disruptions (Gu et al., 2023; Nguyen et al., 2023; Xin and Yang, 2026; Chen et al., 2025), three main challenges persist after a detailed literature review (refer to Section 2). First, very few studies on port resilience consider the diversified internal components and thus fail to acknowledge the ripple effects across different transportation modes such as liner shipping, feeder shipping, railways, trucking, and container yards. Second, the state‐of‐the‐art resilience triangle method, used for calculating performance over time, proves unsuitable for measuring port resilience due to its oversight of crucial criteria. From a port perspective, maintaining satisfactory performance throughout the observation period is critical and should be a primary consideration. Third, port resilience is often assessed using a singular metric, such as handling capacity (Hossain et al., 2019) and demand fulfillment rate (Asadabadi and Miller‐Hooks, 2020). However, given ports’ intricate nature and diversified internal structures, a broader spectrum of indicators should be employed to evaluate port performance. Along with the challenges associated with measuring these indicators, there remains a lack of a methodology capable of integrating them to quantify port resilience holistically.

This study aims to bridge identified gaps by developing a new simulation‐based resilience measurement framework specifically tailored to assess the resilience of multimodal container ports, particularly in response to subsystem disruptions that involve the ripple effect. A system dynamics (SD) model that simulates port operations is developed to achieve this, incorporating feedback loops across five transportation modes: liner shipping, feeder shipping, railway, truck, and container yard. This model leverages real port operational data and historical accident records to generate disruptive scenarios of varying scales and durations. Nine widely recognized port efficiency metrics are identified as Key Performance Indicators (KPIs) to quantify the influence of these disruptions. Building on this foundation, an innovative resilience calculation method is applied to assess port resilience on the basis of selected KPIs, emphasizing the highly recognized and demanded balance among the three essentials of a resilient system: reliability, robustness, and recovery. Building on this approach, a port resilience assessment method utilizing Evidential Reasoning (ER) is introduced, designed to synthesize and generate resilience profiles across different hierarchies. A three‐level criterion framework is adopted to handle inputs from five transportation modes (middle‐level criteria), within each transportation mode, except for the yard, which features two types of KPIs as bottom‐level criteria. Additionally, resilience values obtained from simulation results are translated into a belief structure using the Dempster‐Shafer theory. The criteria are assigned weights on the basis of entropy‐based methods, which consider data variability as an indicator of its significance. The approach is validated via extensive testing from diverse perspectives, with its effectiveness substantiated by experimental, statistical, and correlation analyses. The model's applicability is demonstrated through an adaptable theoretical framework, broad application prospects, and significant practical value. The key contributions of this work are outlined as follows:

1. An original resilience evaluation approach tailored for ports is introduced for broad application across most multimodal container ports. This framework effectively integrates a sequence of modeling techniques, including an SD simulation model, a reliable resilience measurement approach, and ER, to facilitate the systematic evaluation of port resilience in various subsystem disruptions.
2. The operational risks within multimodal ports are analyzed from a micro‐perspective by segmenting the port based on its transportation functions. An SD model, leveraging field investigation and real data, incorporates feedback loops to simulate the ripple effect between these transportation modes, effectively demonstrating the cascade of disruptions within the port.
3. An innovative resilience calculation method is adopted, refining the traditional resilience measurement approach commonly used. This advanced method balances the importance of performance degradation rate, recovery rate, and average performance level, aligning with the criteria necessary for a resilient port.

Specific performance metrics are customized for different transport modes within the port, and their integration through ER enables a systemic quantification of port resilience in response to disruptions. This article is among the first to quantitatively incorporate performance across various transportation modes under disruption into the systematic assessment of port resilience.

The structure of the rest of the article is as follows: Section 2 introduces existing current research on port resilience as well as related methodologies. Section 3 outlines the proposed framework for assessing port resilience, which includes the SD simulation, resilience calculation, and resilience evaluation model. Section 4 presents the experimental results, along with their corresponding analysis and interpretation. Finally, Section 5 summarizes the findings.

## 2 Literature Review

The typical procedure for evaluating system resilience consists of (i) establishing KPIs, (ii) defining resilience metrics (RMs) based on KPIs, and (iii) assessing resilience on the basis of the predefined RMs (Chen et al., 2022). Therefore, the literature review is structured around these three key areas to define the state of the art.

### 2.1 SD in Port Operations and Ripple Effect

In port risk management, simulation methods have attracted considerable attention due to their ability to capture the complex and dynamic nature of transportation systems (Guo et al., 2023; Li et al., 2024). Simulation techniques, such as SD, agent‐based modeling, discrete‐event simulation, graph‐theory‐based simulation, and optimization‐based models, are widely employed to analyze complex system behavior, track system evolution, and support long‐term decision‐making (Ivanov, 2017). Among them, SD stands out for its capacity to model and visualize dynamic systems by mapping causal relationships among variables (Ghadge et al., 2022; Bell et al., 2023). Compared with mathematical approaches that often encounter computational limitations, SD offers a more intuitive and flexible means of capturing both linear and nonlinear dynamics (Er Kara et al., 2021). Furthermore, SD is particularly for assessing resilience under varying disruptive scenarios through parameter adjustments and simulation experiments (Valaei Sharif et al., 2023).

Within the context of port operations, studies have shown that major disruptions lead to prolonged waiting times, underscoring the significance of understanding and mitigating these ripple effects to enhance port operations (Guo et al., 2023). This terminology represents a conceptualization of risks cascading through a network, spreading from one segment to another, and inducing indirect secondary losses due to the interconnectivity and dependency among network elements. This phenomenon is also known as “risk diffusion,” “snowball effect,” “domino effect,” “cascading effect,” and “propagation” (Ghadge et al., 2022). These effects are evident in terms of the frequency of risk events, their impacts, duration, and the scope of the recovery periods (Sokolov et al., 2016).

Among the methods discussed, SD emerged as crucial for studying the ripple effects in large‐scale systems that exhibit complex, multi‐variable interactions (Liu, Wang, et al., 2023). This method addressed the complex interdependencies among port operations, energy, resources (Garbolino et al., 2016), technology, regional economics, and pandemics (Korzebor and Nahavandi 2024; Anderson et al., 2023). For instance, SD was utilized to examine how policy influences the disaster preparedness behaviors among industry actors, revealing a bidirectional relationship between regulatory measures and industry responses (Kwesi‐Buor et al., 2019). Moreover, an SD model was employed to evaluate the effectiveness of three congestion alleviation strategies for dual‐port operations during the COVID‐19 epidemic (Lin et al., 2022). It was also applied to explore the impact of COVID‐19 on shipping and port operations across five subsystems, assessing economic impacts under six scenarios regarding epidemic duration and capacity recovery (Zhou et al., 2022). The effectiveness and versatility of a novel SD model integrating the SEIR epidemiological framework were demonstrated by evaluating container port congestion, using Ningbo Zhoushan Port as a case study (Liu, Wang, et al., 2023). Furthermore, SD was adopted and implemented through a conceptual framework and data‐driven simulation to evaluate the congestion at Shanghai Port (Xu et al., 2021). SD‐based simulations effectively captured time‐dependent factors, including disruption duration escalation, capacity degradation, and recovery processes. For systems characterized by temporal changes in their behavior, SD outperformed other previously utilized simulation methods, as supported by earlier works (Chen et al., 2021; Becerra‐Fernandez et al., 2020). Moreover, the time‐varying outputs derived from SD serve as a foundation for calculating resilience values. On the basis of previous literature analysis, the adaptability and strength of SD in our proposed model were recognized; therefore, SD is selected in this article to establish causal relationships within the port under risk and simulate the ripple effect.

### 2.2 Port Resilience Measurement

Many concepts were adopted to evaluate the performance of transportation systems during exposure to risks, among which the concept of resilience has gained growing scholarly attention recently (Wan et al., 2018). Traditionally, risk is characterized by the combination of severity and its likelihood or frequency. As a result, risk assessment primarily emphasizes the statistical fitting of probabilities and consequences (Choi, 2021). In contrast, resilience assessment takes a more holistic view by evaluating a system's capacity to anticipate, withstand unexpected hazards, preserve essential functions, and recover swiftly. This is particularly relevant for port operations, where the ability to continue functioning during and after disruptions is critical. Strengthening this capacity holds both practical value for operation and theoretical significance.

Consequently, this has resulted in the development of many metrics and indicators (Eisenberg et al., 2019). Summarizing previous studies, RMs are categorized into three types: topological, attribute‐, and performance‐based (Wang and Yuen, 2022), as shown in Table 1.

**TABLE 1:** Comparison of different resilience metrics.

	Advantages	Disadvantages	References
Topological metrics	Simple, suitable for a large‐scale network; Focus on network structure	Sacrifice accuracy; overlooks traffic flow and redistribution; Neglect dynamic features	Bai et al. (2023); Asadabadi and Miller‐Hooks (2020); Dui et al. (2021); Liu, Yang, et al. (2023)
Attribute‐based metrics	Reflect system dynamics; Facilitate comparison across different systems	Definition varies among attributes and requires predefinition; Exclusively focus on specific periods	Gu et al. (2020); Xu et al. (2022); Cheng et al. (2022)
Performance‐based metrics	Reflect system dynamics; Access the performance throughout the entire duration; Standardize different dimensions	Hard to evaluate the instantaneous rate of change	Gu and Liu (2023); Zhen et al. (2022); Zohoori et al. (2023)

Topological metrics originate from graph theory principles (Bai et al., 2023), attribute‐based metrics focus on particular features like recovery speed or efficiency, which may vary on the basis of the chosen attribute, and performance‐based metrics assess resilience in terms of system degradation and restoration throughout the disruption and restoration phase (Cheng et al., 2022). Graphically, performance‐based metrics often measure resilience as the area beneath the performance curve, also known as the “resilience triangle” (Wan et al., 2018). Although specific performance indicators vary significantly across different systems and studies, using performance‐based methods simplifies the selection of appropriate performance indicators. It enables the standardization of metrics across different dimensions, making it a recognized and popular choice in resilience measurement. In this study, disruptions at ports lead to adverse effects on several KPIs. As a result, performance‐based metrics are deemed the most suitable for assessing resilience in this context.

The resilience triangle remains a widely adopted method in performance‐based assessments, which is first introduced as early as 2003 (Bruneau et al., 2003), where resilience is measured by comparing the system's performance after an external shock with its initial level, along with the duration needed for recovery to the original performance level. The understanding of resilience has recently expanded and is now widely used. It no longer merely signifies a return to a stable equilibrium but now encompasses the capacity of socio‐technical systems to sustain a specific mode of operation (Pan et al., 2022). Therefore, a highly resilient system should exhibit (i) a gradual decline in performance (i.e., low degradation rate), (ii) a swift restoration of performance levels (i.e., high recovery rate), and (iii) consistently satisfactory performance throughout the evaluation period (Cheng et al., 2022). The three features are also used as three criteria to measure port resilience in this study. Table 2 provides a comparison of the formulas based on the resilience triangle and their consistency with three fundamental resilience criteria. Specifically, formulations that integrate over the complete disruption and recovery timeline but do not distinguish between degradation and recovery phases (Bruneau et al., 2003; Simonovic and Peck, 2013) fail to satisfy criteria (i) and (ii), as they mix the separate attributes of resilience. Given the complexity of the adopted method, full details are provided in Section 3.2. The relevant notation is provided in Table 3.

**TABLE 2:** Overview of the resilience triangle and its alignment with the resilience definition.

Reference	Formulation of the resilience triangle	Meet the resilience definition		
		(i)	(ii)	(iii)
Bruneau et al. (2003)	$\int_{T_{i}}^{T_{r}}\left(100-P(t)\right)dt$			√
Simonovic and Peck (2013)	$\frac{\int_{T_{i}}^{T_{r}}P\left(t\right)dt}{\int_{T_{i}}^{T_{r}}P_{0}\left(t\right)dt}$			√
Franchin and Cavalieri (2015)	$\frac{\int_{T_{i}}^{T_{r}}P\left(t\right)dt}{P_{0}\left(t\right)\left(T_{i}-T_{r}\right)}$			√
Habibi et al. (2025)	$\frac{\int_{T_{i}}^{T_{r}}P\left(t\right)dt}{T_{i}-T_{r}}$			√
Mugume et al. (2015)	$\frac{\int_{T_{i}}^{T_{r}}\left(P_{0}\left(t\right)-P\left(t\right)\right)dt}{P_{0}\left(t\right)}$			√
Cimellaro et al. (2010)	$\alpha \frac{\int_{T_{i}}^{T_{f}}f\left(t\right)dt}{\Delta T_{f}}+\left(1-\alpha \right)\frac{\int_{T_{f}}^{T_{r}}r\left(t\right)dt}{\Delta T_{r}}$	√	√	
Chanda and Srivastava (2016)	$\frac{\int_{T_{i}}^{T_{r}}\left[P_{0}\left(t\right)-\min\left\{P\left(t\right)\right\}\right]dt}{\int_{T_{i}}^{T_{r}}\left[T_{r}P_{0}\left(t\right)-\min\left\{P_{0}\left(t\right)\right\}\right]dt}$			√
Kadri et al. (2015)	$\frac{\int_{T_{i}}^{T_{r}}P\left(t\right)dt}{\left(T_{r}-T_{i}\right)\int_{T_{i}}^{T_{r}}P_{0}\left(t\right)dt}$			√
Ayyub (2014)	Equation (2) in Section 3.2	√	√	√

**TABLE 3:** Notations of performance indicators employed in resilience quantification.

Notation	Description
$T_{0}$	Time when disruption occurs
$T_{i}$	Time when disruption impact onsets
$T_{f}$	Time when performance degrades to an unacceptable level, upon which the recovery action starts, if applicable
$T_{r}$	Time when system totally recovers
$\Delta T_{f}$	Duration from $T_{i}$ to the moment of lowest performance $T_{f}$
$\Delta T_{r}$	Duration from $T_{f}$ to the moment of total recovery $T_{r}$
$P_{0}\left(t\right)$	System performance level without disruption at time t
P(t)	System performance level under disruption at time t
f(t)	System performance level throughout failure phase at time t
r(t)	System performance level throughout recovery phase at time t

Most traditional resilience triangle methods do not satisfy the first two resilience criteria because they do not separate the degradation phase from the recovery phase. Under such practice, they might equate a robust system with slow recovery to one with poor robustness but high recovery capability. Therefore, this study introduces and validates an improved computational method from previous literature (Ayyub, 2014), refining the original resilience triangle. This method explicitly addresses the three distinct terms: (i) inherent reliability, (ii) average robustness, and (iii) average recovery capacity, aligning more closely with port stakeholders’ expectations for a resilient port. A detailed comparison and interpretation are presented in Section 3.2.

### 2.3 Multiple Indicator Aggregation

Multiple indicator aggregation models serve as crucial instruments for synthesizing diverse structural data when assessing the resilience of systems with multiple indicators (Wen et al., 2024). When applied to port resilience, it allows for the evaluation of an extensive array of factors, surpassing the limitations of concentrating solely on a single aspect of the port, thereby significantly enhancing evaluation quality. In the requirements of our research subject, objectives, and data structure, several commonly used multi‐criteria aggregation methods are reviewed and then compared with the ER. The comparison is summarized in Table 4.

**TABLE 4:** Comparison of other commonly used aggregation methods.

Aggregation methods	Advantage	Disadvantage
Utility Values (weighted sum)	Simple and computationally efficient	Cannot evaluate resilience at the sub‐system level
Analytic Hierarchy Process (AHP);	Clear hierarchical structure	Require strict pairwise‐consistency, which is hard to guarantee when expert opinions are incomplete or conflicting
Fuzzy set (Gu and Liu, 2023)	Handle uncertainty	Assume monotonicity among criteria and do not expose middle layer results
TOPSIS (Wang et al., 2022)	Data‐driven evaluation	Ignore epistemic uncertainty and provide no mechanism for conflict resolution
DEMATEL (Liu, Gu, et al., 2023; Liang et al., 2025)	Reveal causal relationships	Heavily reliant on expert judgment and results, highly dependent on subjective inputs
Bayesian Network (BN) (Liang et al., 2025)	Handle uncertainty	Demand conditional‐independence assumptions and substantial prior data, both impractical in our context

A widely used method for multiple indicator aggregation in ER, proposed by Yang and Singh (1994), is built on the Dempster‐Shafer evidence theory (Dempster, 2008). ER starts by establishing a multi‐level framework comprising multiple KPIs with a hierarchical structure, ranging from top‐level port resilience down to bottom‐level indicators that clearly illustrate the relationships and dependencies between different levels. Information at each level is assessed and processed independently before being aggregated upward. This progressive handling is well suited for aggregating hierarchical criteria of large volumes of scenarios, as it preserves intermediate belief distributions across all levels, ensuring transparency in the identification of resilience at any specific level (Yang and Xu, 2013). In this research, the focus extends beyond solely assessing the overall resilience of the port. It also includes the resilience of various transportation modes due to (i) their significant managerial value and (ii) the dynamic behaviors of them under disruptions, which reveal patterns of ripple effects. Additionally, the aggregated criteria originate from different areas of the port and hence are inherently independent. Advanced ER‐based models were applied in various resilience‐related studies, such as navigational risks in inland waterways (Zhang et al., 2016), port vulnerability (Jiang et al., 2021), and canal vulnerability (Jiang et al., 2023). In comparison, ER yields more objective conclusions than traditional aggregation models, such as AHP and DEMATEL. Consequently, ER is chosen to enable the practical assessment and integration of the resilience of individual transportation mode and their interdependence.

## 3 Methodology

**FIGURE 1:**
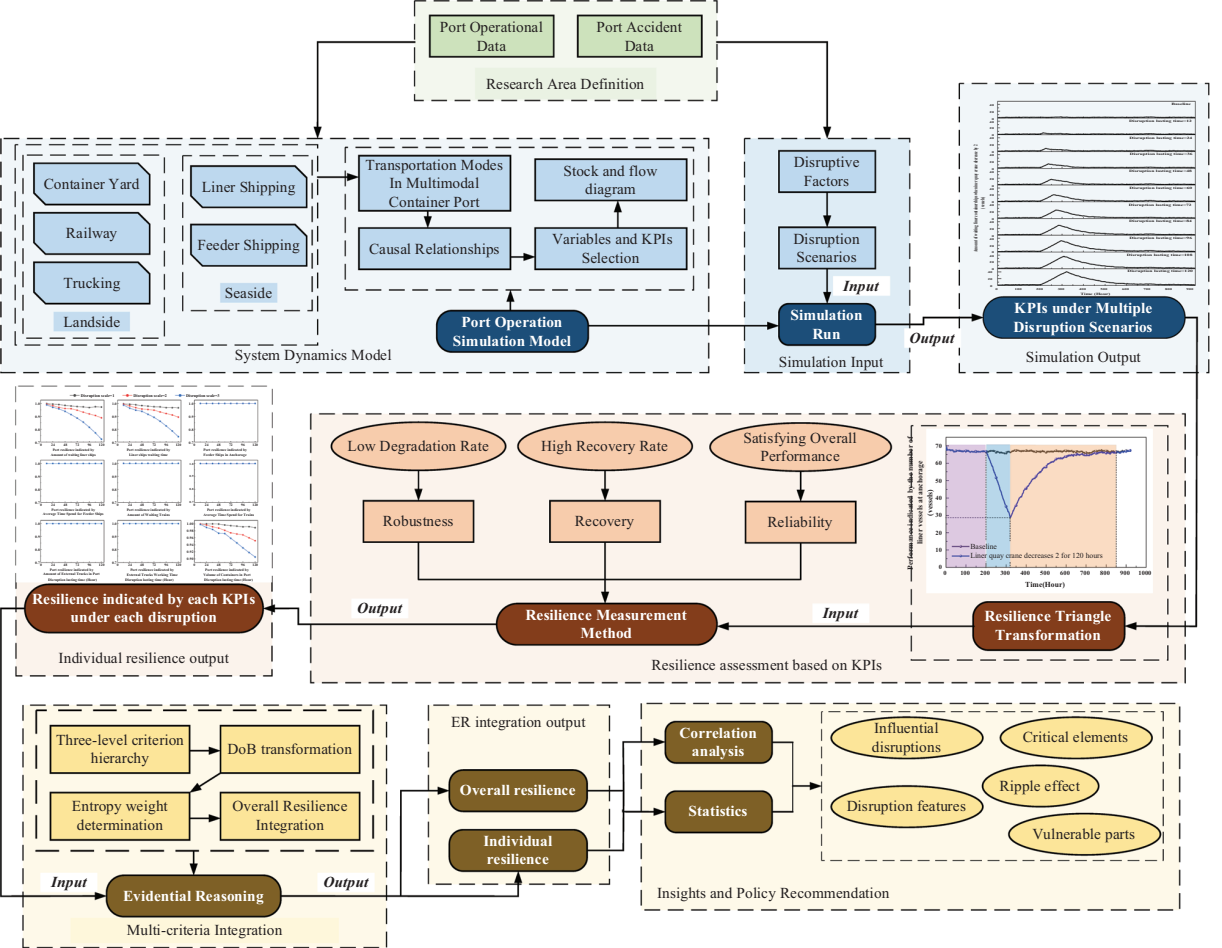
Methodology framework. DoB, Degree of Belief; ER, Evidential Reasoning; KPI, Key Performance Indicator.

A comprehensive description of the proposed methodology is given in this section to assess the resilience of multimodal container ports. External factors, such as economic fluctuations, technological advancements, and regulatory changes, are treated as upstream causes, and after their impact on internal disruptions is clearly identified, they can be integrated into the current model framework to evaluate the influence of external factors on port resilience. As shown in Figure 1, the methodology begins with simulating port operations under normal conditions using SD in Section 3.1. Subsequently, disruptive scenarios are introduced by adjusting specific variables, identified as disruption variables. The variation in value ranges of disruption variables simulates different levels of impact, with each scenario defined by disruption type, intensity, and duration. The selection of disruption variables and their corresponding value ranges is justified by historical accident reports from port operations and expert input, as detailed in Section 4.1.1. These disruption scenarios are then used as simulation inputs to generate time dependent outputs of port performance, measured through various KPIs. Given the multimodal structure of ports, each mode is evaluated using dedicated KPIs. For each disruptive scenario, the value of a given KPI under both normal and this disruptive scenario serves as input for calculating a resilience value based on the formula in Section 3.2. This process is repeated across all selected KPIs, generating a set of individual resilience values that reflect different aspects of port performance. The ER model receives these inputs and combines them to derive an overall resilience score, which serves as the final output and represents the port's integrated performance under the given disruptive scenario. The subsequent sections will outline the procedures for developing the simulation model, calculating resilience for each KPI, and assessing port resilience in Section 3.3.

### 3.1 Port Operations Simulation

Modern equipment, layout designs, and operational protocols of container ports are highly standardized. Therefore, research findings from several typical multimodal ports support the development of operational processes for a conceptual, generic container port. Among all port operations, loading and unloading, container storage, container collection (export), and distribution (import) are deemed essential for ensuring smooth operations. Thus, this article's development of the port operation simulation model primarily focuses on these critical activities.

The development process for the simulation model comprises the following steps: (i) establishing modeling hypotheses, (ii) identifying the simulation procedures, (iii) selecting input variables, and (iv) selecting output variables (KPIs).

#### 3.1.1 Simulation Hypothesis

A series of hypotheses is formulated, grounded in field observations and relevant scholarly works, before introducing the simulation model (Liu, Wang, et al., 2023; Li, Haralambides, et al., 2022; Jin et al., 2021).

1. The multimodal container port incorporates four modes of transportation: liner shipping, feeder shipping, railway transport, and trucking. Liner and feeder shipping are modeled as distinct components, reflecting their unique operational demands and functions in container supply chains. Liner containerships, typically operating over long distances, call at hub ports, whereas feeder containerships connect these hubs to regional feeder ports.
2. Liner containerships deliver inbound containers and subsequently distribute them via feeder containerships, trains, and trucks. In return, outbound containers are gathered in storage yards via feeders, trains, and trucks then loaded onto liner containerships for export.
3. Internal trucks facilitate intra‐port movements, supporting operations across all transportation modes within the port.
4. Yard truck operations include internal and external trucks, with internal trucks prioritized to ensure consistent operational efficiency, independent of external truck numbers. Therefore, internal truck waiting times are typically excluded from consideration.
5. Resource constraints, such as limited cranes, berths, and trucks, hinder smooth container handling processes.
6. Disruptions within ports often spread through the interactions facilitated by internal trucks.

#### 3.1.2 Simulation Procedures

In SD, causal loop diagrams that depict causal relationships illustrate the principles guiding the simulation procedures. This subsection aims to clarify the operational logic of multimodal container ports by providing a detailed description of the causal loop diagram's structure. Beyond the four primary transportation modes, the port incorporates centralized facilities dedicated to the temporary storage, sorting, and transshipment of containers, which occupy a significant expanse of the port's spatial allocation. Therefore, the SD model is developed modularly, comprising five key transportation modes (subsystems): liner shipping, feeder shipping, container yard, truck, and railway.

**FIGURE 2:**
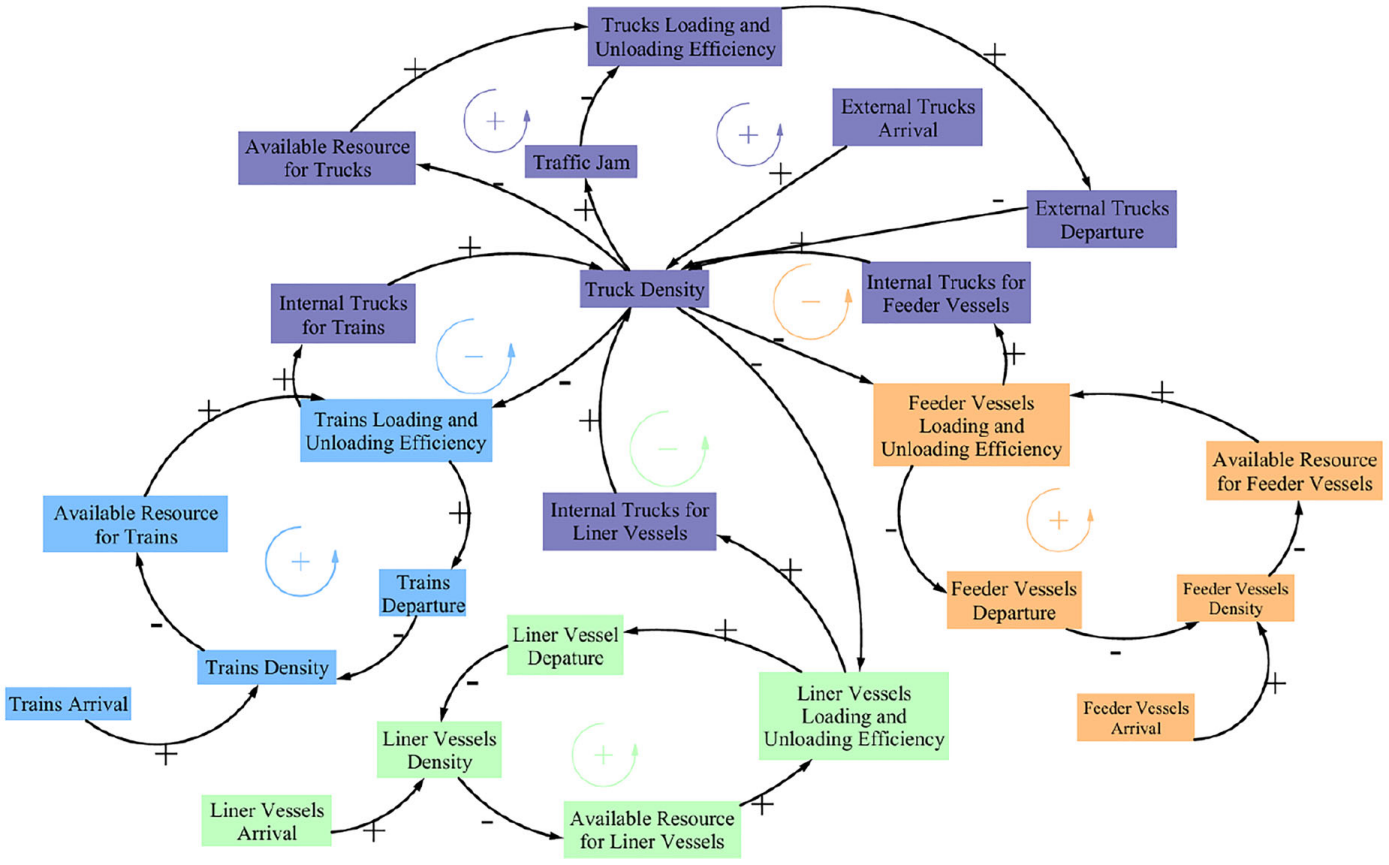
Causal loop diagram.

**FIGURE A1:**
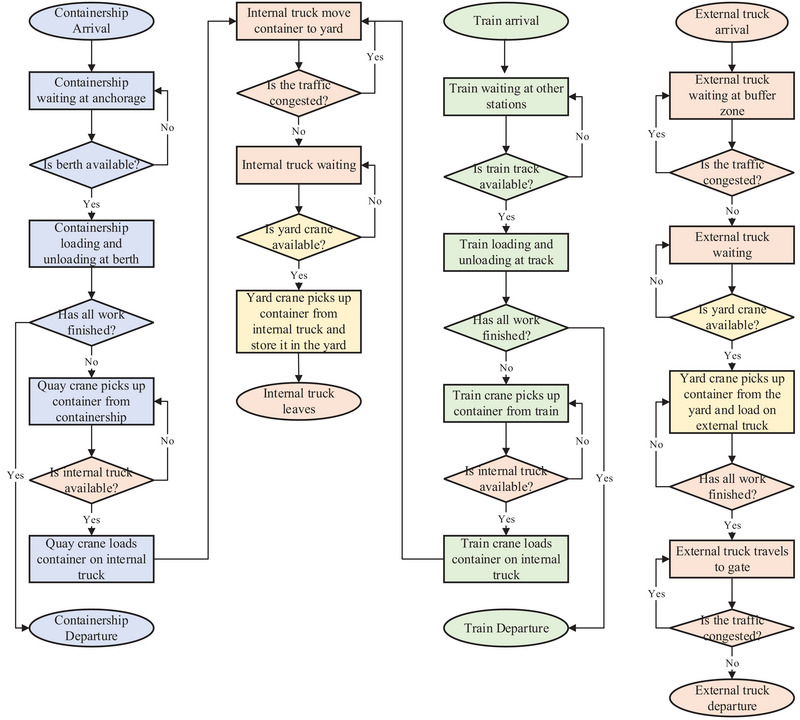
Operational flow in the port.

**FIGURE A2:**
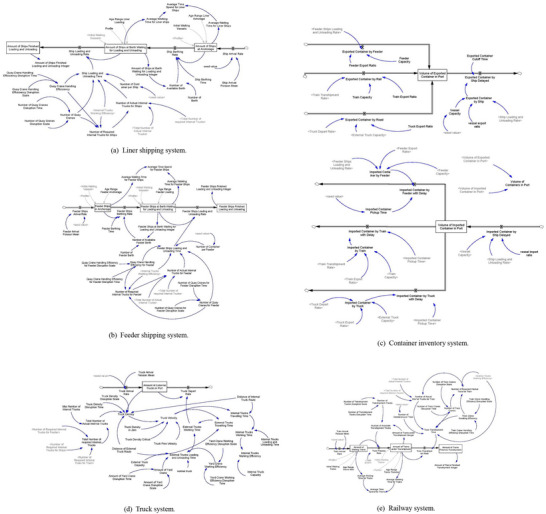
Stock‐flow diagram of a multimodal container port. (a) Liner shipping system; (b) Feeder shipping system; (c) Container inventory system; (d) Truck system; (e) Railway system.

On the basis of field investigation and prior studies, the causal loops for each subsystem are identified and classified by transportation modes in Table 5. Notably, L2 (liner shipping), L4 (feeder shipping), and L6 (railway) all include truck related variables associated with L7 and L8 (truck operations) due to the role of internal trucks as a key connector. Moreover, as shown in Figure 2, truck density serves as a central node linking multiple transportation modes. The detailed operational workflow for these transportation modes is presented in Figure A1. On the basis of field investigation and prior studies, the causal loops for each subsystem are identified and classified by transportation modes in Table 5, highlighting the modular structure. However, L2 (liner shipping), L4 (feeder shipping), and L6 (railway) all include truck related variables associated with L7 and L8 (truck operations). This is due to the role of internal trucks as a key connector between various port areas. Reflected in our model, in Figure 2, truck density is positioned as a central node connecting multiple transportation modes. Additionally, for each transportation mode, the components in the causal loop diagram are translated into specific variables, supported by constants, parameters, intermediate variables, and shadow variables. These elements are interconnected through mathematical equations, enabling simulation, as shown in Figure A2. Additionally, it is crucial to recognize that these interconnections not only highlight the functional dependencies across subsystems but also provide potential pathways for the propagation of ripple effects within the port system.

**TABLE 5:** Details of the loops of each subsystem.

Notation	Subsystem	Detail
L1	Liner shipping	Liner vessels density $\;\bar{\to }\;$ Available resource for each liner vessel $\overset{+}{\to }$ Liner vessels loading and unloading efficiency $\overset{+}{\to }$ Liner vessels departure rate $\bar{\to }\;$ Liner vessels density
L2	Liner shipping	Liner vessels loading and unloading efficiency $\overset{+}{\to }$ Internal trucks for liner vessels $\overset{+}{\to }$ Truck density $\;\bar{\to }$ Liner vessels loading and unloading efficiency
L3	Feeder shipping	Feeder vessels density $\;\bar{\to }\;$ Available resource for each feeder vessel $\overset{+}{\to }$ Feeder vessels loading and unloading efficiency $\overset{+}{\to }$ Feeder vessels departure rate $\bar{\to }\;$ Feeder vessels density
L4	Feeder shipping	Feeder vessels loading and unloading efficiency $\overset{+}{\to }$ Internal trucks for feeder vessels $\overset{+}{\to }$ Trucks density $\bar{\to }$ Feeder vessels loading and unloading efficiency
L5	Railway	Trains density $\;\bar{\to }\;$ Available resource for each train $\overset{+}{\to }$ Trains loading and unloading efficiency $\;\overset{+}{\to }$ Trains departure rate $\bar{\to }\;$ Trains density
L6	Railway	Trains loading and unloading efficiency $\overset{+}{\to }$ Internal trucks for train $\;\overset{+}{\to }$ Trucks density $\bar{\to }$ Trains loading and unloading efficiency
L7	Truck	Trucks density $\;\bar{\to }\;$ Available resource for each external truck $\overset{+}{\to }$ External trucks loading and unloading efficiency $\overset{+}{\to }$ External trucks departure $\bar{\to }\;$ Trucks density
L8	Truck	Trucks density $\;\overset{+}{\to }$ Traffic jam $\bar{\to }$ External trucks loading and unloading efficiency $\;\overset{+}{\to }$ External trucks loading and unloading time $\;\bar{\to }\;$ Trucks density

The general causal loop diagram demonstrates how various factors interact with each other. Directional arrows are used to visualize causal links, where the origin indicates the cause and the arrowhead denotes the resulting effect. These links are further classified by “+” or “−” signs, indicating whether the relationship is reinforcing or counteracting. A “+” suggests a positive correlation, whereas a “−” shows a negative effect. When multiple factors form a loop, these symbols also indicate whether the loop acts as a positive reinforcing loop or a negative balancing loop, as shown in Figure 2. The underlying mechanisms in each feedback loop are explained in the figure.

L1 represents a feedback structure of the liner shipping subsystem, illustrating how resource availability impacts the waiting metric of liner containerships. Abundant berthing resources, along with necessary loading and unloading equipment, enhance operational speed, increase containership departure rates, and consequently reduce the queue of waiting containerships. L2, also within the liner shipping subsystem, focuses on a feedback loop featuring internal trucks. Container transfer operations depend on the synchronized movement of internal trucks between the quay side and the yard side, making the availability of internal trucks critical for efficient operations. Insufficient internal truck support directly undermines the efficiency of liner container ships’ loading and unloading processes. Conversely, increased internal trucks might cause heightened congestion, leading to delays, longer turnaround times, and decreased operational efficiency.

L3 (feeder shipping), L5 (railway), and L7 (trucking) follow the same logic as L1. L4 (feeder shipping) and L6 (railway) follow the same logic as L2.

L8 illustrates the feedback loop in which truck density influences the efficiency of both external and internal truck loading and unloading operations. Truck operations at a container port distinguish between (i) external trucks, which handle deliveries and pickups between the port and hinterland, and (ii) internal trucks, which move containers within the port among storage yards and other locations. High truck density leads to traffic congestion, which extends turnover time and reduces the efficiency of both types of trucks. Consequently, the departure rate of external trucks decreases, further exacerbating the congestion in the container yard.

#### 3.1.3 Variable Selection

The stock‐flow diagram is derived from the causal loop diagram through a careful selection of variables that accurately represent the logical relationships outlined previously. In the simulation model, variables are categorized as input and output variables (KPIs), both of which are time dependent. Input variables comprise constants and intermediate parameters necessary for model formulation and execution. Output variables, represented as KPIs, are obtained after simulation and used to evaluate port performance. This section begins by detailing the rationale for selecting input and output variables, followed by a description of the key variables adopted for performance evaluation. Finally, it features a modular illustration of the stock‐flow diagram, encompassing five subsystems and various quantitative variables, as shown in Figure A2. The relevant variables used in these models are outlined in Table 6. For details on each variable and its calculation method, please refer to Table A1.

**TABLE 6:** Input variables.

Subsystem	Factor
Container Inventory (Li et al., 2023; Jin et al., 2021; Lin et al., 2022)	Containers inventory level, export and import flow rate, and container cutoff time
Liner shipping (Zhou et al., 2022; Liu, Wang, et al., 2023; Xu et al., 2021)	Arrival and departure rate of liner vessels, availability of loading and unloading resources (berths, cranes), handling efficiency (cranes, trucks, berthing process), and capacity (workload of containers)
Feeder shipping (Lee and Jin 2013; Emde and Boysen 2016)	Arrival and departure rate of feeder vessels, availability of loading and unloading resources (berths, cranes), handling efficiency (cranes, trucks, berthing process), and capacity (workload of containers)
Truck (Liu, Wang, et al., 2023; Li, Haralambides, et al., 2022; Li et al., 2018)	Arrival and departure rate of external trucks, availability of loading and unloading resource (internal trucks, yards cranes), handling efficiency (cranes), traveling speed, capacity (workload of containers), and traveling distance
Railway (Xu et al., 2021; Schulz et al., 2021; Liu, Wang, et al., 2023)	Arrival and departure rate of trains, availability of loading and unloading resources (tracks, cranes), handling efficiency (cranes, trucks), and capacity (workload of containers)

This model's primary objective is to evaluate the impact of potential disruptions on port efficiency. Therefore, KPIs for port operations are established on the basis of industry standards and expert insights. Additionally, the chosen output variables are widely recognized metrics for evaluating container port efficiency, supported by the sources referenced in Table 7.

**TABLE 7:** Main performance indicators.

Category	KPIs	References
Liner shipping	Amount of waiting liner vessels	Liu, Wang, et al. 2023; Zhou et al. 2022
	Liner vessels dwell time	
Feeder shipping	Amount of waiting for feeder containerships	Emde and Boysen 2016; Jin et al. 2021
	Feeder vessels dwell time	
Railway	Amount of waiting trains	Schulz et al. 2021
	Trains dwell time	
Trucking	Amount of external trucks in the port	Liu, Wang, et al. 2023; Xu et al. 2021; Li et al. 2018
	Turnaround time of external trucks	Li et al. 2018; Sun et al. 2022
Container yard	The inventory level of containers in the port	Liu, Wang, et al. 2023; Lin et al. 2022; Xu et al. 2021

Abbreviation: KPIs, Key Performance Indicators.

### 3.2 Resilience Measurement Method

**FIGURE 3:**
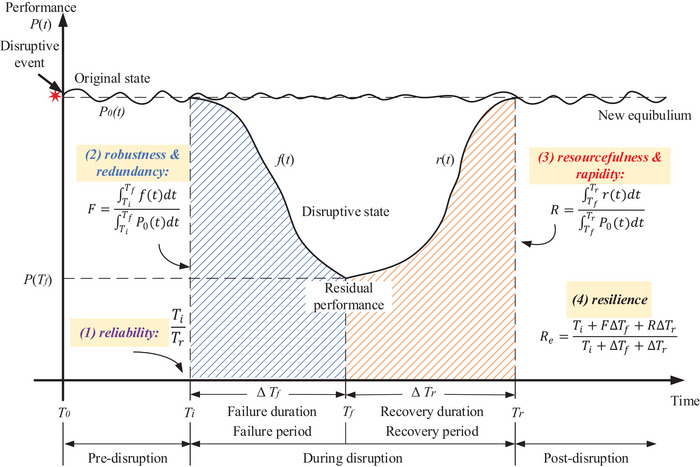
Resilience quantification using performance indicators.

On the basis of the simulation in Section 3.1, port performance under normal conditions is presented as a time‐dependent curve $P_{0}\left(t\right)$ in Figure 3. By adjusting the disruption variables, the disrupted performance curve P(t) is also generated. Both curves are illustrated in Figure 3 at the same time. Although the performance curves represent the temporal dynamics of port operations, they do not provide a comprehensive measure of the port's overall ability to resist and recover from disruptions. To overcome this limitation, the resilience triangle method is applied to convert the time‐dependent performance curve into a single, integrated resilience value.

As explained in the previous section, the commonly used resilience triangle method, which no longer fully meets current needs, is depicted in the following equation. The notations involved are listed in Table 3 in Section 2.2:

(1) $$R=\frac{\int_{T_{i}}^{T_{r}}P\left(t\right)dt}{\int_{T_{i}}^{T_{r}}P_{0}\left(t\right)dt}$$

where R is the ratio of disruptive and normal performance during the disruptive and recovery periods. It is explained in detail that various configurations of P(t) and $T_{r}$ can yield identical resilience values R, yet they may exhibit distinct resilience features (Sun et al. 2024). The misinterpretation is due to two inherent flaws. First, the traditional method does not formally define nor incorporate the performance turning point (i.e., the point when performance reaches the bottom and the recovery process begins) into the assessment, despite this point being visually identifiable and significant in shaping the performance curve. Consequently, the performance degradation and recovery phases are treated as a single continuous period. Second, this prevents the independent evaluation of robustness (before the lowest point) and recovery (after the lowest point).

To improve Equation (1), this work adopts a novel resilience assessment that incorporates multiple perspectives, as shown in Equation (2) and Figure 3 (Ayyub, 2014). This method follows a structured and theoretically grounded approach, which has been recognized within the research community (Cheng et al., 2022, Sun et al., 2024). Yet, its application in port resilience evaluations remains unexplored:

(2) $$\begin{array}{c} R_{e}=\frac{T_{i}+F\Delta T_{f}+R\Delta T_{r}}{T_{i}+\Delta T_{f}+\Delta T_{r}}\\ F=\frac{\int_{T_{i}}^{T_{f}}f\left(t\right)dt}{\int_{T_{i}}^{T_{f}}P_{0}\left(t\right)dt}\\ R=\frac{\int_{T_{f}}^{T_{r}}r\left(t\right)dt}{\int_{T_{f}}^{T_{r}}P_{0}\left(t\right)dt} \end{array}$$

where the failure profile F is considered a measure of robustness and redundancy, and the recovery profile R is considered a measure of resourcefulness and rapidity. Upon decomposing R, it is divided into three parts:

1. The normalized time required for a disturbance to take effect $\frac{T_{i}}{T_{r}}$. In theory, it indicates the proportion of the incident to the entire duration. However, in practice, the onset of effects from each disturbance varies. This factor quantifies the time required for the effects of a disturbance to take hold.
2. The product of normalized failure performance F and normalized failure duration $\frac{\Delta T_{f}}{T_{r}}$.
3. The product of normalized recovery performance $R_{c}$ and normalized recovery duration $\frac{\Delta T_{r}}{T_{r}}$.

The notations involved in Figure 3 are listed in Table 3 in Section 2.2. Under each disruption scenario, port performance is evaluated from the perspective of each KPI, resulting in a performance curve P(t) as illustrated in Figure 3. In this method, P(t) is further divided at time $T_{f}$, when performance reaches its minimum, into two parts: f(t) representing performance degradation from the disruption onset to $T_{f}$ and r(t) for the recovery phase from $T_{f}$ until the port performance returns to its original level. Under port resilience framework, $R_{e}$ is decomposed into three indicators: (i) inherent reliability, (ii) average robustness, and (iii) average recovery capacity. By extending previous models to incorporate all three core dimensions of resilience, this approach allows for an adequate characterization and summarization of the resilience of a multimodal container port throughout the entire disruptive period.

### 3.3 Port Resilience Evaluation Model Based on ER

The port's operational performance is assessed across multiple dimensions using the KPIs listed in Table 7. According to Section 3.2, the time independent assessment is converted into a set of resilience values corresponding to multiple KPIs. Therefore, a key methodological challenge is to integrate these KPI‐based resilience values to derive both overall port resilience and the resilience specific to each transportation mode. To address this, resilience values are calculated for each KPI under a given disruptive scenario. The ER approach then aggregates these into an overall value while retaining individual characteristics. The integration process is achieved through the following steps from Section 3.3.1 to Section 3.3.4, respectively:

1. **Formulation of hierarchy structure**: The hierarchy is formulated with three levels of criteria to account for multiple KPIs (bottom‐level criteria) across transportation modes (middle‐level criteria) and overall port resilience (top‐level criteria).
2. **Resilience value and belief structure**: The steps introduced in Section 3.2 determine the resilience value for each disruptive scenario. Subsequently, this score is mapped into a belief distribution.
3. **Weight determination**: The influence of low‐level criteria on higher level criteria is determined on the basis of the information they encompass.
4. **Overall resilience integration**: Employing the established criteria hierarchy, the model calculates the port's overall resilience on the basis of belief distributions and determined weights.

#### 3.3.1 Part 1: Formulation of Hierarchy Structure

**FIGURE 4:**
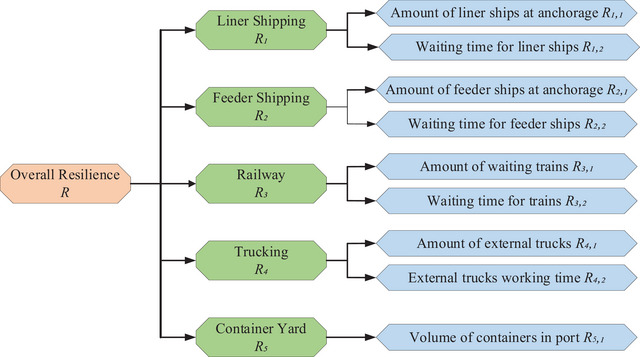
The three‐level hierarchy for port resilience evaluation.

A hierarchical structure with three levels of criteria is constructed, as depicted in Figure 4. At the top level, the overall resilience of the port is denoted by R. At the second level, the port is segmented on the basis of five distinct transportation modes: liner shipping $R_{1}$, feeder shipping $R_{2}$, railway $R_{3}$, trucking $R_{4}$, and container yard $R_{5}$. Apart from $R_{5}$, each mode comprises two types of KPIs, labeled as $R_{j,k}$.

#### 3.3.2 Part 2: Resilience Value and Belief Structure

Due to the shift to individual KPI rather than a hierarchical structure in this section, the KPIs previously denoted as $R_{j,k}$, are now defined as $E=\{e_{1},e_{2},\text{…},e_{i},\text{…},e_{I}\},$ indexed by i∈[1,9], where each $e_{i}$ corresponds to $R_{j,k}.\;A=\left\{a_{1},a_{2},\text{…},a_{l},\text{…},a_{L}\right\}$ defines the alternative vector, containing a total of L disruptive scenarios. $H=\{H_{1},H_{2},\text{…},H_{n},\text{…},H_{N}\}$ represents the set of resilience grades with N indicating the total number of grades. The resilience score for KPI $e_{i}$ under a disruptive scenario *l* is given by $\alpha_{i}\left(a_{l}\right)$, and the procedure for obtaining it is outlined in Section 3.2. The range for each resilience grade is determined by the distribution of resilience values across all KPIs. For example, the four grades of R are defined as ${\{}^{\prime }strong^{\prime }{,}^{\prime }moderate^{\prime }{,}^{\prime }weak^{\prime }{,}^{\prime }minimal^{\prime }\;\}\;$ according to previous research (Xu et al., 2023; Gu and Liu 2023). When $\alpha_{i}\left(a_{l}\right)\;$ falls between two predefined grades, a linear distribution method is applied. The belief distribution $S(e_{i}\left(a_{l}\right))$, evaluated from the perspective of $e_{i}$ under the specific disruption scenario $a_{l}$, is presented in the following equation:

(3) $$S\left(e_{i}\left(a_{l}\right)\right)=H_{n},\beta_{n,i}\left(a_{l}\right),\text{…},H_{N},,\beta_{N,i}\left(a_{l}\right)n=1,2,\text{…},N$$

where N=4, $H_{n}$ represents the n th evaluation grade, and $\beta_{n,i}\left(a_{l}\right)$ indicates the Degree of Belief (DoB) from the perspective of $e_{i}$, the resilience of the port is evaluated as $H_{n}$ under the disruptive scenario $a_{l}$. Significantly, $0\leq \beta_{n,i}\left(a_{l}\right)\leq 1$ and $\sum_{n=1}^{N}\beta_{n,i}\left(a_{l}\right)\leq 1.$ In this study, $\sum_{n=1}^{N}\beta_{n,i}\left(a_{l}\right)=1$, which ensures that the assessment is complete, and there is no residual mass unassigned to any grade, that is, $\beta_{n,i}\left(a_{l}\right)\equiv 0$ for any $e_{i}.$ This subjectivity is captured through the belief distribution framework, allowing for a thorough consideration of all available information.

#### 3.3.3 Part 3: Weight Determination

The resilience value of a specific KPI may demonstrate a biased influence on the overall evaluation outcomes of port resilience. Therefore, it is essential to effectively incorporate information from multiple KPIs to bridge this critical gap. This scenario exemplifies a classical multi‐criteria assessment where it is critical to allocate weights to each information source (KPIs of the port) based on their significance. The entropy weight method is employed to determine the relative importance of different KPIs in the resilience evaluation. This data‐driven approach offers a practical alternative to expert‐based judgment. Given that all KPIs are evaluated under the same set of disruption scenarios, a KPI that exhibits greater sensitivity to these disruptions implies a stronger impact on the affected corresponding transportation mode. Such aspects should therefore be given higher priority in the resilience evaluation framework to ensure that their influence on overall port resilience is accurately represented (Feng et al., 2023; Wang et al., 2022; Shakibaei et al., 2024; Liu et al., 2017).

Typically, the entropy weighting method begins with data standardization to neutralize the influence of measurement units. However, this initial step is unnecessary when resilience values fall within the same range, as introduced in Section 3.2. The probability set $P_{i}\;$ is computed on the basis of the proportion of the l th disruptive scenario in the i th KPI, which is denoted by $p_{i}\left(a_{l}\right)$. Therefore, the probability used in the entropy calculation is obtained by

(4) $$P_{i}=\left\{p_{i}\left(a_{1}\right),p_{i}\left(a_{2}\right),\text{…},p_{i}\left(a_{L}\right)\right\}=\left\{\frac{\alpha_{i}\left(a_{1}\right)}{\sum_{l=1}^{L}\alpha_{i}\left(a_{1}\right)},\text{…},\frac{\alpha_{i}\left(a_{L}\right)}{\sum_{l=1}^{L}\alpha_{i}\left(a_{L}\right)}\right\}$$

The entropy of the *i*th KPI, denoted as $Q_{i}$, is then determined on the basis of the definition of information entropy:

(5) $$Q_{i}=-k\sum_{l=1}^{L}p_{i}\left(a_{1}\right)\ln\left(p_{i}\left(a_{1}\right)\right)$$

(6) $$k=\frac{1}{lnL}$$

wherek is a constant associated with sample size L. A larger $Q_{i}$ means the ith KPI has more entropy, which suggests that it offers less useful information. Therefore, the entropy weight $w_{i}$ of KPI $e_{i}$ is determined by

(7) $$w_{i}=\frac{d_{i}}{\sum_{i=1}^{I}d_{i}}$$

where $d_{i}=1-Q_{i}$ is the information utility value and the entropy‐based weight $w_{i}$ for each KPI is derived by normalizing its corresponding $d_{i}$.

To capture cumulative effects and highlight extreme values, the weight assigned to each transportation mode is determined by summing all associated weights of KPIs.

#### 3.3.4 Part 4: Overall Resilience Integration

On the basis of the belief distribution (Section 3.3.2) and weights (Section 3.3.3), the ER approach is applied to aggregate the values of KPIs from multiple transportation modes across the port, thus constructing a final belief distribution indicating the influence level of disruption. The basic probability mass $m_{n,i}$, representing the belief degree to which KPI $e_{i}$, supports the hypothesis that its upper level transportation mode is assigned to grade $H_{n}$:

(8) $$m_{n,i}=w_{i}\beta_{n,i}n=1,2,\text{…},N,i=1,2,\text{…},I$$

(9) $$m_{H,i}=1-\sum_{n=1}^{N}w_{i}\beta_{n,i}i=1,2,\text{…},I$$

where $m_{H,i}$ denotes the unallocated probability mass not attributable to any specific evaluation grade $H_{n}$ by KPI $e_{i}$. This residual is separated into two components ${\bar{m}}_{H,i}$ and ${\tilde{m}}_{H,i}$, which are obtained by

(10) $${\bar{m}}_{H,i}=1-w_{i}i=1,2,\text{…},I$$

(11) $${\tilde{m}}_{H,i}=w_{i}\left(1-\sum_{n=1}^{N}\beta_{n,i}\right)i=1,2,\text{…},I$$

where ${\bar{m}}_{H,i}$ is bounded by the inherent weight of the criterion $e_{i}$, revealing how much other indicators influence the evaluation; ${\tilde{m}}_{H,i}$ results from incomplete assessment information.

According to the hierarchy structure in Figure 4, the basic probability mass from the bottom‐level $R_{j,k}$ is first aggregated to the middle‐level $R_{h}$ using the recursive ER algorithm. This approach is then repeated until aggregation reaches the top‐level R. The probability mass $m_{n,I(i)}$ quantifies the degree to which the ith KPI within the set $E(e_{i})$ endorse the evaluation grade $H_{n}$, whereas $m_{H,i}$ unallocated probability mass not attributable to any specific evaluation grade by these KPIs. Initially, $m_{n,I(i)}=m_{n,1}$ and $m_{H,I(i)}=m_{H,1}$. The combined probability mass for i=1,2,…,L are derived as follows:

(12) $$m_{n,I\left(i+1\right)}=K_{I\left(i+1\right)}\left(m_{n,I\left(i\right)}m_{n,i+1}+m_{n,I\left(i\right)}m_{H,i+1}+m_{H,I\left(i\right)}m_{n,i+1}\right)$$

(13) $${\tilde{m}}_{H,I\left(i+1\right)}=K_{I\left(i+1\right)}\left({\tilde{m}}_{H,I\left(i\right)}{\tilde{m}}_{H,i+1}+{\bar{m}}_{H,I\left(i\right)}{\tilde{m}}_{H,i+1}+{\tilde{m}}_{H,I\left(i\right)}{\bar{m}}_{H,i+1}\right)$$

(14) $${\bar{m}}_{H,I\left(i+1\right)}=K_{I\left(i+1\right)}\left({\bar{m}}_{H,I\left(i\right)}{\bar{m}}_{H,i+1}\right)$$

(15) $$K_{I\left(i+1\right)}={\left(1-\sum_{t=1}^{N}\sum_{j=1,j\neq t}^{N}m_{t,I\left(i\right)}m_{j,i+1}\right)}^{-1}$$

where $m_{n,I(i+1)}$ denotes the degree of support for $H_{n}$ when recursively combining the DoB of the *i*th KPI with (i+1)th KPI. $K_{I(i+1)}$ serves as the normalization factor. After aggregating all the information, the combined DoB is obtained as follows:

(16) $$\beta_{n}=\frac{m_{n,I\left(L\right)}}{1-{\bar{m}}_{H,I\left(L\right)}}n=1,2,\text{…},N$$

(17) $$\beta_{H}=\frac{{\tilde{m}}_{H,I\left(L\right)}}{1-{\bar{m}}_{H,I\left(L\right)}}$$

where $\beta_{n}$ represents the aggregated DoB that a resilience value is evaluated as $H_{n}$, and $\beta_{H}$ is the aggregated DoB that remains unassigned to any specific evaluation grade, indicating the level of uncertainty in the assessment. Port resilience under the scenario $a_{l}$ is characterized by the subsequent belief‐based distribution:

(18) $$S\left(R\left(a_{l}\right)\right)=\left\{\left(H_{n},\beta_{n}\left(a_{l}\right)\right),n=1,2,\text{…},N;\left(H_{N},\beta_{N}\left(a_{l}\right)\right)\right\}$$

where $S(R(a_{l}))$ denotes the belief distribution of the overall port resilience under the disruption scenario $a_{l}$. Through a linear transformation, a single overall resilience value $R(a_{l})$ is derived. The same approach is used to evaluate the resilience of different transport modes:

(19) $$R\left(a_{l}\right)=\sum_{n=1}^{N}\frac{n-1}{N-1}\beta_{n}\left(a_{l}\right)$$

## 4 Experimental Results

### 4.1 Experiment Setup and Design

#### 4.1.1 Disruption Settings

To validate the performance and practicality of the methodology, it is essential to select a port with significant international influence, advanced multimodal transport facilities, and representative risk profiles. Therefore, a globally recognized container port with an integrated multimodal transport system is selected for empirical analysis. This port serves as a key hub for container transshipment through waterways, highways, and railways, making it an ideal site for analyzing the ripple effects of interconnected systems. Moreover, given that most container ports adhere to a standardized layout and utilize consistent equipment, the nature of accidents tends to be uniform (Bogalecka and Dąbrowska, 2023; Lu and Borgonovo, 2023), which ensures that the insights gained from the study apply to other ports.

The data for this study are grounded on three primary data sources: (i) A field investigation was conducted at the investigated port in September 2023, during which the real‐time operational data were collected and firsthand insights into daily port activities were gained. These findings help define the logical flow of container port operational processes. (ii) Expert interviews are involved throughout the model construction, including the variable selection (refer to Table 6) and identification of KPIs (refer to Table 7), the interaction among variables and subsystems, the selection of disruptive scenarios (refer to Table 8), and the model validation. (iii) Port accident reports from 1998 to 2021 are analyzed to extract disruption patterns and quantify corresponding scenarios. Recent findings indicate that port equipment failures, container structural damage, and traffic accidents are the most frequently occurring port accident types. From the perspective of consequences, equipment damage, personal injury, and traffic‐related incidents are the most common causes of severe damage. A recently published related study provides additional detailed information for data collection and model validation (Zhang et al., 2025).

**TABLE 8:** Scenario settings.

Disrupted transportation mode	Disrupted component	Disruption scale	Disruption lasting time
Liner shipping	Quay cranes	1,2,3	12,24,36,48,60,72,84,96,108,120
Feeder shipping	Quay cranes	1,2	12,24,36,48,60,72,84,96,108,120
Railway	Train cranes	1,2,3	12,24,36,48,60,72,84,96,108,120
Container yard	Yard cranes	4,8,12,16,20,24	12,24,36,48,60,72,84,96,108,120
	Traffic density	0.2,0.4,0.6,0.8,1.0	12,24,36,48,60,72,84,96,108,120

Having identified the major accidents, the next steps involve defining the magnitude of these incidents from two perspectives: their severity (Chen et al., 2022) and duration (Cao and Lam, 2018). Drawing on actual practices and expert opinions, specific settings of the accidents are determined, as outlined in Table 8. Subsequently, several disruption scenarios are established. The outputs of these scenarios serve as a basis for evaluating the consequences of disruption, providing the foundation for assessing port resilience.

On the basis of the identified disruption scenarios, simulations of port disruptions are conducted. A total of 190 different disruption scenarios are analyzed, including 30 in liner shipping, 20 in feeder shipping, 30 in railway, and 110 in container yards. To direct the readers’ attention to the implications of resilience values under different scenarios, the term “sensitivity analysis” is not explicitly used in the subsequent analysis. Nevertheless, the approach of varying parameter values adopted here falls within the scope of sensitivity analysis. Disruptions should be introduced after the system has stabilized to avoid initial biases. To ensure this, a warm‐up period of 200 h is applied. This duration is based on the observation that the values of all KPIs stabilize after approximately 200 h under normal operating conditions. Additionally, to gather sufficient data and observe long‐term behavior patterns while ensuring representative results considering the liner shipping schedule, the simulation spans 920 h. Each scenario is repeated 30 times to calculate average values and reduce noise. Subsequently, the resilience values are calculated using the methods detailed in Sections 3.2 and 3.3.

#### 4.1.2 Validation

This subsection validates the proposed methodology from two aspects: the performance of the SD simulation model and the overall resilience assessment framework, respectively. The reliability and accuracy of the resilience measurement method have been verified and recognized through comparisons with traditional approaches (Cheng et al., 2022; Sun et al., 2024); further validation is not conducted here.

##### 4.1.2.1 Validation for the SD Model

To validate the SD model, established methods widely recognized in previous studies were employed (Sterman, 2010; Qudrat‐Ullah, 2012). The specific procedures, their suitability, and the corresponding results are documented in a recently published related work (Zhang et al., 2025). Overall, all tests were successfully passed.

Table 9 presents the validation results for behavior reproduction, where the simulation results are compared with historical data. Due to the lack of data, five factors represent the five transportation modes. Prior literature suggests that variations within ±10% were considered acceptable for model validation (Liu, Wang, et al., 2023). The findings demonstrate that the simulation results align closely with the actual operations of the port.

**TABLE 9:** Behavior reproduction validation result.

Variables	Average estimate	Average actual	Relative variation (%)
Number of external trucks in port	500.81	500	0.162
Inventory level of containers in the port	102,107.5	110,000	7
Number of liner containerships in port	12.89 vessels	14 vessels	7.92
Number of feeder containerships in port	2.21 vessels	2 vessels	10.5 (accepted after rounding up)
Number of trains in the port	2.87 trains	3 trains	4.3

##### 4.1.2.2 Validation of the Overall Framework

Second, the entire framework, which includes an SD model, a resilience calculation method, and the ER model, is validated. In previous studies, most integrated frameworks only validated the simulation part, neglecting the validation of the entire framework (Feofilovs and Romagnoli, 2021; Blouin et al., 2024). Given the absence of systematic methods for validating such a framework, a limitation acknowledged by scholars (Chin et al., 2009), the extreme condition approach from SD validation is adopted. This approach examines whether the overall framework can effectively capture the dynamic behaviors of port resilience changes. Extreme scenarios, as detailed in Table 10, are designed for this verification. The results confirm that under extreme disruption conditions, resilience values decrease dramatically, as expected. Under these extreme conditions, the resilience of the associated KPIs often reaches a global minimum, sometimes nearing zero, as shown in Table 11.

**TABLE 10:** Scenario settings for extreme condition test.

Transportation mode	Base value	Extreme scenario name	Extreme value
Liner shipping	Liner quay crane = 6	ES 1	Liner quay crane = 1, liner quay crane disruption time = 200
Feeder shipping	Quay crane handling efficiency for feeder = 30	ES 2	Quay crane handling = 10, quay crane handling efficiency for feeder disruption time = 240
Railway	Number of transshipment tracks = 4	ES 3	Number of transshipment tracks disruption scale = 1, number of transshipment tracks disruption time = 200
Trucking	Max number of internal trucks = 60	ES 4	Max number of internal trucks = 20

**TABLE 11:** Extreme condition test results.

Resilience value	ES 1	ES 2	ES 3	ES 4
$R_{1,1}$	0.418	0.986	0.986	0.733
$R_{1,2}$	0.517	0.994	0.994	0.731
$R_{1}$	**0.001**	0.567	0.567	0.119
$R_{2,1}$	0.956	0.580	0.956	0.730
$R_{2,2}$	0.983	0.579	0.983	0.734
$R_{2}$	0.309	**0.005**	0.309	**0.001**
$R_{3,1}$	1.000	1.000	0.696	0.581
$R_{3,2}$	0.948	0.948	0.698	0.432
$R_{3}$	0.631	0.631	**0.001**	**0.001**
$R_{4,1}$	1.000	1.000	1.000	1.000
$R_{4,2}$	1.000	1.000	1.000	1.000
$R_{4}$	1.000	0.949	1.000	1.000
$R_{5,1}$	0.466	0.719	0.885	1.000
$R_{5}$	0.466	0.719	0.885	1.000
R	0.240	0.287	0.317	0.491

### 4.2 Experimental Results

In this section, the overall port resilience under different disruption scenarios (see Table 8) is first analyzed, identifying incident types significantly affecting overall resilience and their statistical characteristics. Second, the impact of these disruptions on various transportation modes is examined in detail. Lastly, through a correlation analysis, the article explores the characteristics of ripple effects within the port, thereby demonstrating the effectiveness of this study in understanding and managing port risks.

#### 4.2.1 Results of SD Simulation

**FIGURE 5:**
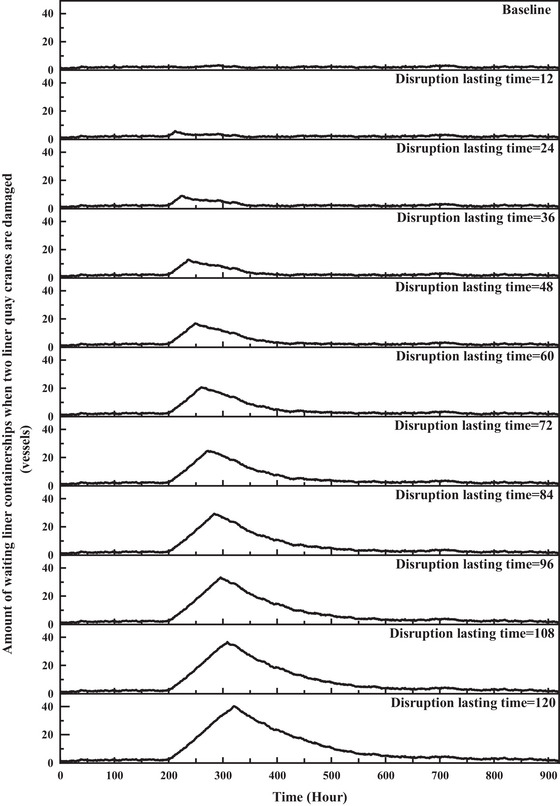
SD simulation results of the number of waiting liner containerships when two liner quay cranes are damaged under different disruption durations.

The SD model generates time series data for nine KPIs under various disruption scenarios characterized by different types, magnitudes, and durations of disruptions. Given the extensive dataset, Figure 5 depicts the variance trend of liner containerships waiting at anchorage due to the damage of two liner quay cranes. The duration of the disruption extends from 0 h (baseline, no disruptions) up to 120 h.

#### 4.2.2 Results of Resilience Indicated by KPIs

**FIGURE 6:**
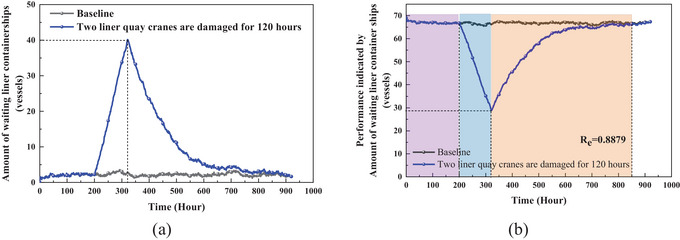
Illustration of resilience measurement on the basis of performance indicator: The number of waiting liner containerships when two liner quay cranes are damaged for 120 h. (a) Time‐series performance under disruption; (b) Normalized resilience triangle and calculated resilience value.

Considering the extensive dataset, which includes over 200 scenarios and 9 KPIs, an example involving a 120‐h failure of 2 liner quay crane is chosen to demonstrate the process of resilience calculation. A slice of Figure 5 is shown in Figure 6a. The data are normalized on the basis of the global maximum disruptive magnitude to construct resilience triangles, as shown in Figure 6b. Subsequently, the resilience value is calculated using Equation (2).

**FIGURE 7:**
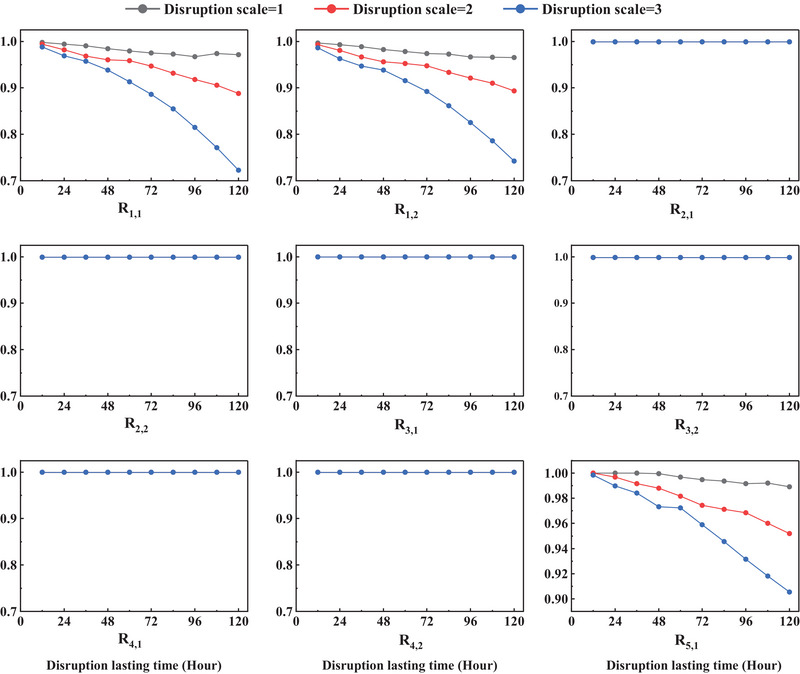
Resilience calculation results due to different scales of quay crane disruptions across different durations indicated by nine KPIs.

Through the above steps, the resilience values indicated by all KPIs are calculated. Due to the vast amount of data, the results under the liner quay crane damage scenarios are selected as an example, as shown in Figure 7. In the case of quay crane disruptions, the scales correspond to the removal of 1, 2, or 3 cranes, indicating progressively severe operational impacts. The same scaling principle is applied to the remaining disruption types in Table 8.

#### 4.2.3 Overall Port Resilience Results

##### 4.2.3.1 Entropy Weight Results

Using the resilience values, the weights for each KPI and each transportation mode are determined on the basis of the entropy weight method described in Equations (4)–(7), as shown in Table 12. From the perspective of transportation modes, container storage is of paramount significance, followed by liner and feeder shipping. Rail and road transport carry the lowest weight, aligning with typical industry patterns. At the bottom level, the weight of KPIs shows minimal variation, indicating that they are nearly equally important to their respective higher level indicators.

**TABLE 12:** Entropy weight results (three‐decimal precision).

Resilience	Weight	Resilience	Weight	Resilience	Weight	Resilience	Weight	Resilience	Weight
$R_{1}$	0.198	$R_{2}$	0.219	$R_{3}$	0.127	$R_{4}$	0.138	$R_{5}$	0.319
$R_{1,1}$	0.485	$R_{2,1}$	0.508	$R_{3,1}$	0.475	$R_{4,1}$	0.582	$R_{5,1}$	1
$R_{1,2}$	0.515	$R_{2,2}$	0.492	$R_{3,2}$	0.525	$R_{4,2}$	0.418		

##### 4.2.3.2 Overall Port Resilience Under Different Disruption Events

**FIGURE 8:**
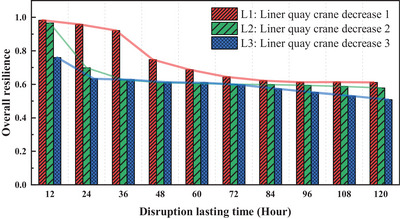
Impact of liner quay crane disruptions on overall resilience.

**FIGURE 9:**
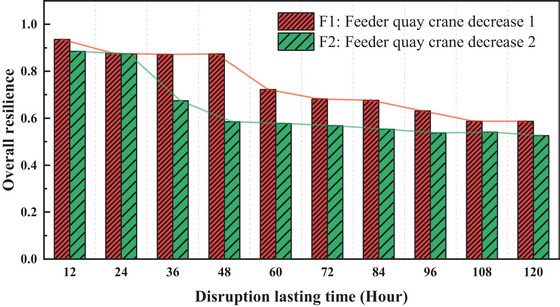
Impact of feeder quay crane disruptions on overall resilience.

**FIGURE 10:**
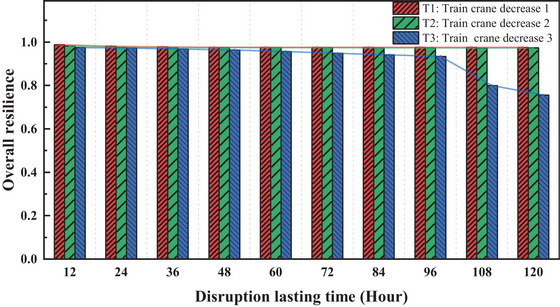
Impact of train crane disruptions on overall resilience.

**FIGURE 11:**
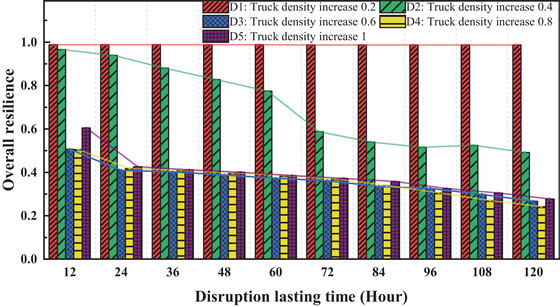
Impact of traffic jams on overall resilience.

**FIGURE 12:**
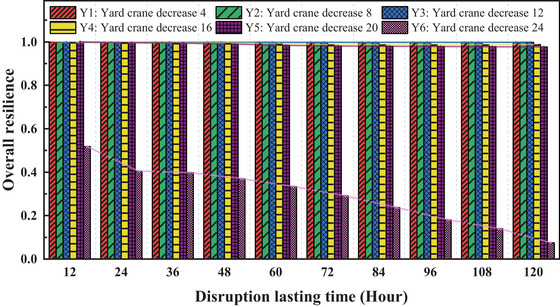
Impact of yard crane disruptions on overall resilience.

Using resilience values and weights as inputs, the overall port resilience value across different disruption scenarios is derived through ER. As the primary objective of this research is to develop and validate a systematic framework for assessing port resilience, each disruption scenario is predefined and treated deterministically, without incorporating uncertainty. This assumption applies to the results presented in Figures 8, 9, 10, 11, 12. Figure 8 illustrates the overall port resilience in response to different severities of liner quay crane disruptions. Generally, as disruption escalates (i.e., with longer durations and a fewer operational quay cranes for liner shipping), the port resilience value decreases accordingly. The most pronounced decline in resilience occurs at 48 h for L1, 24 h for L2 and L3, indicating a potential breaking point. After 60 h, the variation in resilience values among L1, L2, and L3 becomes minimal, and the decline rate due to prolonged disruptions also diminishes. This suggests that the disruption duration and magnitude have a limited influence on total resilience, likely because the port system's recovery capabilities reach a saturation point. However, before 60 h, the resilience values drop markedly as the severity and duration of disruptions increase, demonstrating a heightened sensitivity. Therefore, more attention should be focused on managing the severity and duration of disruptions within the first 60 h, where they tend to have a more pronounced effect.

Figure 9 illustrates the overall port resilience in response to various feeder quay crane incidents. Like liner quay cranes, resilience values experience substantial decreases due to disruptions within the first 60 h, underscoring the importance of this period for effective mitigation and the restoration of normal operations. Additionally, the disparity between scenarios F1 and F2 is initially small (12–24 h), widens during the 36–48‐h period, and then narrows, emphasizing the criticality of the 36–48‐h‐period in managing and mitigating the disruption effects.

Figure 10 shows overall port resilience under different severities of incidents involving train cranes. Throughout the study period, resilience levels for the T1 and T2 scenarios remain constant, with only the T3 scenario experiencing significant fluctuations when the disruption persists for 96 h. This suggests that, compared to incidents involving liner and feeder quay cranes, accidents in rail operations have a lesser impact, possibly due to the higher self‐recovery capacity or redundancy inherent in railway systems.

Figure 11 depicts the overall port resilience under different traffic congestion scenarios. Under the D1 scenario, the port's resilience remains largely stable, indicating that this congestion level does not significantly impact normal port operations and remains within acceptable limits. In the D2 scenario, resilience exhibits a step‐wise decline, with a significant drop occurring around the 60‐h mark, followed by stabilization. This suggests that this duration represents a critical threshold for port resilience under D2‐level congestion. Under scenario D2, resilience gradually diminishes over time. This declining trend disappears at D3, transitioning to consistently low resilience values that remain nearly constant over time, a pattern that also extends to D4 and D5. Moreover, the resilience levels at D5 are slightly higher than those at D3 and D4, possibly due to disruptions’ complex and nonlinear effects on the overall port system.

Figure 12 illustrates the overall resilience values in the event of yard crane failures at the container yard. As a container block is typically served by a group of yard cranes performing container handling tasks, the failure of a single crane is limited. Therefore, scenarios simulating the complete failure of all crane groups within a block are considered. Under scenarios Y1, Y2, Y3, Y4, and Y5, the number of yard crane failures and the durations have a minor impact on resilience. However, in scenario Y6, when the number of malfunctioning yard cranes exceeds 24, there is a substantial decline in port resilience, which further decreases as the disruption time increases. In practice, ensuring that the number of non‐operational yard cranes remains below 20 is crucial to maintain normal operations at the port.

**FIGURE 13:**
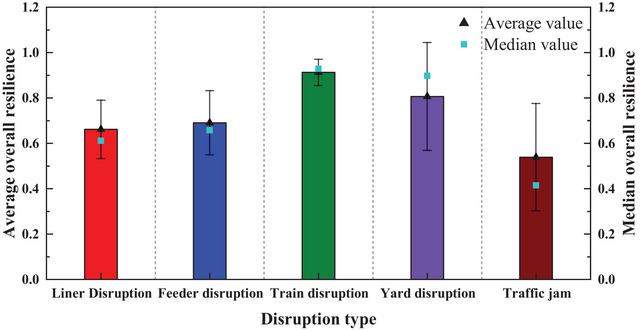
Average, error, and median value for overall port resilience under different types of disruptions.

Figure 13 presents the statistical characteristics of overall port resilience, including mean, error bar, and median values across different types of disruptions. Traffic jams have the most significant impact on port resilience, followed by liner quay crane incidents, with train crane failures having the least impact. This analysis provides port managers with a clear ranking of the severity of different incidents, enabling them to develop tailored response strategies accordingly. The error bar results indicate significant fluctuations in resilience due to yard crane failures and traffic congestion, reflecting the high sensitivity of the system to the degree and duration of these disruptions. The difference between the mean and median values, coupled with the length of the error bars, suggests the presence of outliers. This indicates that the resilience scores in specific disruption categories, such as liner disruption and traffic jam, include outliers significantly lower than most of the data, which may require special attention.

#### 4.2.4 Subsystem Resilience Under Different Disruptive Scenarios

**FIGURE 14:**
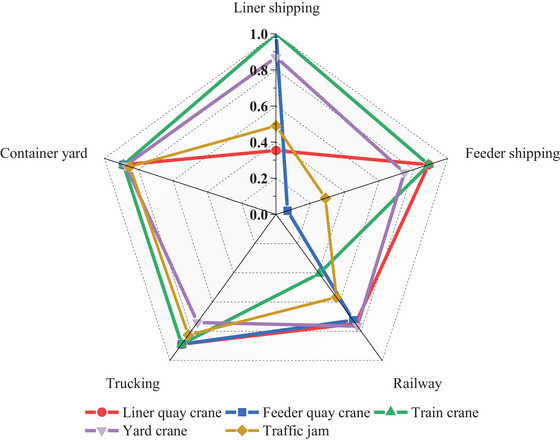
Subsystem resilience values under different disruptions.

Figure 14 illustrates the average resilience values across five transportation modes (i.e., $R_{1},R_{2},R_{3},R_{4},R_{5}$) under different types of disruptions. Each axis represents a specific transportation mode, whereas each colored line corresponds to a distinct type of disruption. The values along the axes indicate the resilience scores. The following analysis is conducted from two perspectives: (i) the comparative resilience of different transportation modes under the same disruption scenario (i.e., along a single colored line), and (ii) the resilience of a given transportation mode under different types of disruptions (i.e., along a single axis). On the basis of these analyses, resource allocation can be strategically prioritized before, during, and after disruptions, and it also facilitates the identification of areas that are resilient and vulnerable.

First, the impacts of a specific disruption type on different transportation modes are revealed. Liner quay crane disruptions have the most significant impact on the liner shipping, with resilience values around 0.3, followed by the railway, whereas others are less affected. This suggests a strong interdependence between liner and railway operations, with the railway particularly susceptible to such disruptions. Feeder quay crane disruptions predominantly affect feeder shipping operations, reducing resilience values to below 0.1, while having a minimal impact on others. Train crane failures primarily influence railway operations, where resilience values approximate 0.4, but their impact is less notable in other operations. Yard crane failures exhibit slight variation across transportation modes, maintaining resilience values above 0.7. Traffic jams significantly disrupt the liner, feeder, and railway operations, underscoring the essential role of container truck operations in linking seaside and railway activities. The impact on the truck operations is moderate, with the least influence on the container yard.

Second, the following analysis examines the resilience values of a specific transportation mode under various disruptions. The liner shipping records the lowest resilience when affected by liner quay crane disruptions, dropping below 0.4, with traffic jams also causing substantial reductions; however, it remains higher than against other disturbances. Similarly, the resilience of feeder shipping operations drops below 0.1 during its corresponding incidents, with traffic jams as the next most disruptive, highlighting operational similarities between liner and feeder shipping. The railway operations experience a resilience value of around 0.4 (train crane accidents), 0.6 (traffic jams), and 0.8 (other disruptions). The truck operations consistently exhibit resilience between 0.7 and 0.8 across all disruptions, with yard crane accidents posing the most significant risk. The container yard demonstrates exceptional resilience across all disruptions, indicating a robust capacity to withstand risks. In summary, seaside shipping operations are the most vulnerable parts of the port.

#### 4.2.5 Correlation Analysis

**FIGURE 15:**
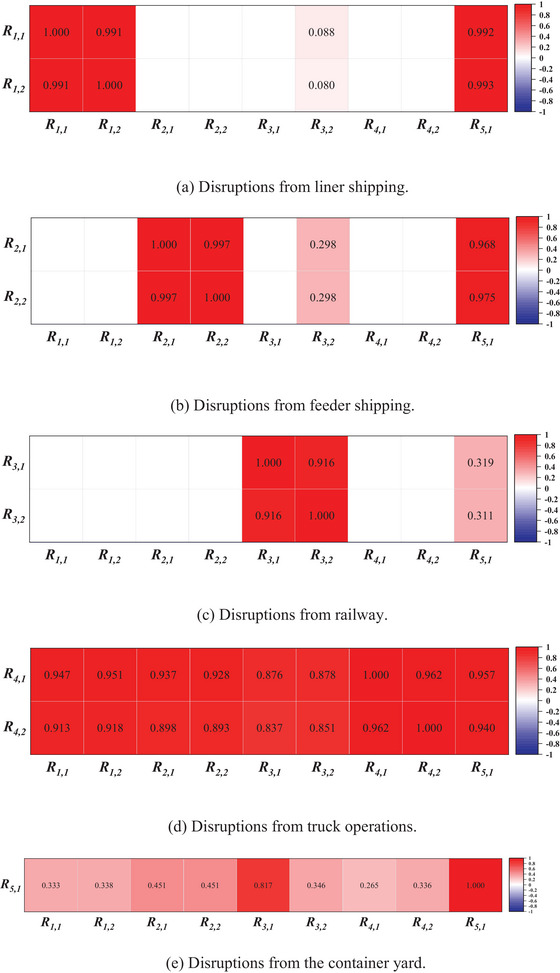
Interdependencies among resilience values from various KPIs of the port. (a) Disruptions from liner shipping; (b) Disruptions from feeder shipping; (c) Disruptions from railway; (d) Disruptions from truck operations; (e) Disruptions from container yard.

Figure 15 illustrates the correlation analysis results between different resilience values represented by different KPIs. Liner shipping disruptions in Figure 15a and feeder shipping disruptions in Figure 15b extend their impact to the container yard. However, disruptions in the railway operations only affect the container yard minimally, as shown in Figure 15c. Additionally, as shown in Figure 15d, the resilience values in the truck operations show strong correlations with those from all other transportation modes, indicating that disruptions originating from here in this subsystem propagate throughout the entire port, leading to widespread efficiency declines. Notably, in Figure 15e, the container yard is particularly correlated with the number of trains waiting at the port, highlighting that container backlogs significantly impair railway operational efficiency, but not the other way around. This effect extends to feeder shipping, underscoring the critical role of rail and waterborne feeder transport in the container port's logistics network. It is important to emphasize that the observed correlations between the RMs of different transport subsystems reflect the dependencies among system ability, but they do not indicate the temporal sequence or lag in disruption propagation. Thus, such analysis provides insight into association, rather than causation or timing of ripple effects.

### 4.3 Implications

In this section, the implications of this research will be discussed from three perspectives: implications on theory, practice, and policymaking.

From a theoretical standpoint, this work advances the current literature by addressing several critical gaps: (i) It challenges the conventional view in port risk management that obscures the understanding of ripple effects among interconnected transportation modes by enriching the literature based on a multimodal examination of port disruptions; (ii) it refines resilience measurement by clearly distinguishing between robustness and recovery capabilities; (iii) it innovatively proposes a framework for port resilience assessment by integrating SD, the resilience triangle, and ER.

From a practical standpoint, the theoretical framework, which accounts for the ripple effects that significantly impact real‐world practices and adheres to a multimodal layout, aligns with actual operational needs. Consequently, this framework is adaptable to ports featuring similar multimodal transshipment systems, offering data‐driven, practical, and reliable guidance for maritime decision‐makers. On the basis of historical data, the framework objectively measures KPIs, the resilience of a specific transportation mode, and overall port resilience under various risk scenarios. Additionally, the correlation analysis enables a clear identification of accident propagation patterns and resulting ripple effects. In summary, this approach could provide relevant stakeholders with quantitative data on the resilience of their operations and equip safety managers and risk analysts with a tool to understand the ripple effects within ports transparently.

From a policy perspective, the proposed methodology has broad applicability and practical value. Its implementation can help port operators, government agencies, and regulators enhance risk prevention during the preparation stage, control risk propagation during disruptions, and accelerate recovery after incidents. In turn, this approach can mitigate the adverse impacts on shipping companies, cargo owners, port authorities, and especially on port workers and unions through the following:

1. This study demonstrates that traffic congestion and disruptions to quay cranes handling liner containerships result in the most substantial decline in resilience. Accordingly, policymakers and port managers should prioritize these areas in their prevention and emergency response strategies, including routine maintenance, inventory of critical spare parts, redundancy in key resources, and investment in intelligent traffic control systems.
2. Resilience patterns under different disruption magnitudes and durations help identify intervention thresholds. In general, this study suggests that the first 60 h after a disruption are the most critical period for resilience recovery. This calls for setting a tailored response time point for each disruption type. Besides, real‐time monitoring and active alert systems can help mitigate ripple effects.
3. Analyzing resilience across transportation modes helps identify vulnerable subsystems and develop targeted strategies based on their exposure profiles. For instance, feeder shipping is primarily affected by direct risks. It requires direct, mode‐specific protection, whereas yard and liner operations are more susceptible to indirect ripple effects and thus require strategies against indirect disruptions.
4. By analyzing the correlation of various KPIs under disruptions, this study identifies patterns of ripple effects within the port. Our findings indicate that disruptions originating on the seaside propagate to the yard side, and disruptions on the yard side affect the entire port. Therefore, policies should target these critical transmission points by enhancing coordination protocol mechanisms, such as regulating container flow between the seaside and yard side during peak hours, adjusting yard storage strategies, and managing internal port traffic conditions.

## 5 Conclusion and Future Work

This study introduces a framework that incorporates SD simulation, resilience assessment method, and ER to examine the resilience of multimodal ports in the face of operational disturbances. It segments ports on the basis of their transportation functions to examine the ripple effects across these modes. Its main contributions include (i) a micro perspective SD simulation tailored for multimodal container ports; (ii) a novel resilience calculation method that distinctly captures the features of disruptive and recovery periods; (iii) an ER approach capable of integrating interconnected and contradictory indicators; (iv) an applicable framework that guides users in conducting their assessments to enhance global port resilience ultimately. Experimental results provide insights into how different transport subsystems and the entire port respond to operational disruptions, as well as the spreading patterns of ripple effects between these modes.

The proposed framework is computationally efficient. However, integrating three components requires data synchronization and coordination, which substantially increases modeling complexity as the number of disruption scenarios grows. To mitigate this, future work may employ machine learning techniques to approximate the relationship between disruption scenarios and resilience values without the need for repeated simulations. Given the unpredictability of risks and limited data, the framework cannot capture all possible disruption scenarios. Future studies will aim to identify the influence of external factors in advance and incorporate a broader range of risk scenarios. Moreover, the current correlation analysis does not consider time delays between disruptions and their impacts, nor concurrent disruption events. Future extensions could address multiple concurrent risks, conduct sensitivity analyses over a wider parameter space, and model ripple effects with lagged causal relationships.

## Appendix A

### A.1

The operational workflow of the different transportation modes within the port is outlined below, with different colors representing different modes. As the operational processes for feeder shipping and liner shipping are identical, they are combined for presentation purposes. Figure A1

Elaborating on the causal loop diagram, the stock‐flow diagram is also developed in a modular manner, encompassing five subsystems and various quantitative variables, as shown in Figure A2.

#### A.1.1

**TABLE A1:** Notation used in the simulation model.

	Notation	Variable	Unit	Estimation	Source
Liner shipping system	$\lambda_{l}$	Ship arrival rate	Vessels/Hour	Poisson (1.5)	Statistics representation derived from real data
	$B_{l}$	Number of available berth	Vessel	16	Real data
	$E_{l}$	Number of quay cranes	Cranes	6	Real data
	$C_{l}$	Number of container per ship	Containers	Random uniform (1000, 3000)	Statistics representation derived from real data
	$\eta_{l}$	Quay crane handling efficiency	Containers/Hour	30	Real data
	$D_{lb}$	Ships berthing time	Hour	1	Real data
	$D_{ll}$	Ship loading and unloading time	Hour	Variable	$D_{ll}=\max\left(\frac{C_{l}}{\eta_{l}E_{l}},\frac{C_{l}}{AI_{l}\eta_{i}}\right)$
	$\mu_{l}$	Ships loading and unloading rate	Vessels/Hour	Variable	$\mu_{l}=\{\begin{array}{c} \frac{L_{l}}{D_{ll}},L_{l}>0\\ 0,\;else \end{array}$
	$L_{l}$	Amount of liner ships at berth loading and unloading	Vessels	Variable	$L_{l}=\int P_{l}-\mu_{l}$
	$A_{l}$	Amount of ships at anchorage	Vessels	Variable	$A_{l}=\int \lambda_{l}-P_{l}$
	$B_{al}$	Number of available berth	Berth	Variable	$B_{al}=B_{l}-L_{l}$
	$P_{l}$	Ships berthing rate	Vessels/Hour	Variable	$P_{l}=\{\begin{array}{c} \frac{\min(B_{al},A_{l})}{D_{lb}},\;B_{al}>0,A_{l}>0\\ 0,\;else \end{array}$
Trucking system	$\lambda_{l}$	Truck arrival rate	TEU/Hour	Poisson (500)	Statistics representation derived from real data
	$s_{t}$	Distance of external truck route	km	5	Real data
	$s_{i}$	Distance of internal truck route	km	3	Real data
	$\rho_{j}$	Truck density in jam	Trucks/km	60	Average real data
	$v_{f}$	Truck free velocity	km/Hour	30	Average real data
	$D_{il}$	Internal trucks loading and unloading time	Hour	0.05	Averaged real data
	$E_{y}$	Amount of yard cranes	Cranes	48	Real data
	$\eta_{y}$	Yard crane working efficiency	Containers/Hour	30	Real data
	$C_{t}$	External trucks capacity	Containers/Trucks	2	Real data
	$C_{i}$	Internal trucks capacity	Containers/Trucks	2	Real data
	$E_{i}$	Max number of internal trucks	Trucks	60	Real data
	$a_{t}$	Intercept coefficient	Dimensionless	−1.5	Real data
	$b_{t}$	Slope coefficient	Dimensionless	0.0045	Real data
	$D_{tl}$	External truck loading and unloading time	Hour	Variable	$D_{tl}=\frac{1}{a_{t}+b_{t}E_{y}\eta_{y}}C_{t}$
	$D_{tt}$	External truck travelling time	Hour	Variable	$D_{tt}=\frac{s_{t}}{v}$
	$D_{tw}$	External truck working time	Hour	Variable	$D_{tw}=D_{tt}+D_{tl}$
	v	Truck velocity	km/Hour	Variable	$v=v_{f}\ln\left(\frac{\rho_{j}}{\rho }\right)$
	$A_{t}$	Amount of external trucks in port	Trucks	Variable	$A_{t}=\int \lambda_{t}-\mu_{t}$
	ρ	Truck density	Trucks/km	Variable	$\rho =\frac{A_{t}+AI_{i}}{\max(s_{t},s_{i})}$
	$\mu_{t}$	Truck depart rate	Trucks/Hour	Variable	$\mu_{t}=\{\begin{array}{c} \frac{A_{t}}{D_{tw}},\;A_{t}>0\\ 0,\;else \end{array}$
	$RI_{m}$	Number of required interna trucks for transportation mode m	Trucks	Variable	$RI_{m}=\frac{\eta_{m}E_{m}}{\eta_{i}},\forall m=\left\{l,f,r\right\}$
	$RI_{i}$	Total number of required Internal trucks	Trucks	Variable	$RI_{i}=\sum_{m=l,f,r}RI_{m}$
	$AI_{i}$	Number of actual internal trucks	Trucks	Variable	$AI_{i}=\min\left(E_{i},RI_{i}\right)$
	$AI_{m}$	Number of actual internal trucks for transportation mode m	Trucks	Variable	$AI_{m}=\frac{AI_{i}RI_{m}}{RI_{i}},\forall m=\left\{l,f,r\right\}$
	$\eta_{i}$	Internal truck working efficiency	Containers/Hour	Variable	$\eta_{i}=\frac{C_{i}}{D_{iw}}$
	$D_{it}$	Internal truck travelling time	Hour	Variable	$D_{it}=\frac{s_{i}}{v}$
	$D_{iw}$	Internal truck working time	Hour	Variable	$D_{iw}=D_{it}+D_{il}$
Container import and export system	α	Exported containers cutoff time	Hour	72	Averaged real data
	β	Imported containers pickup time	Hour	RANDOM UNIFORM (72, 120)	Statistics representation derived from real data
	$x_{f}$	Feeder ships export ratio	Dimensionless	0.5	Averaged real data
	$x_{r}$	Train export ratio	Dimensionless	0.5	Averaged real data
	$x_{t}$	Trucks export ratio	Dimensionless	0.5	Averaged real data
	$x_{l}$	Liner ships export ratio	Dimensionless	0.35	Averaged real data
	$y_{f}$	Feeder ships capacity	Containers/Vessels	Random Uniform (500, 1000)	Statistics representation derived from real data
	$y_{r}$	Trains capacity	Containers/Trains	Random Uniform (100, 150)	Statistics representation derived from real data
	$y_{l}$	Liner ships capacity	Containers/Vessels	Random Uniform (1000, 3000)	Statistics representation derived from real data
	$O_{m}$	Exported container by transportation mode m	Containers	Variable	$O_{m}=x_{m}\mu_{m}y_{m},\forall m=\left\{l,f,r,t\right\}$
	$Q_{m}$	Imported container by transportation mode m	Containers	Variable	$Q_{m}=1-x_{m}\mu_{m}y_{m},\forall m=\left\{l,f,r,t\right\}\;\;$
	$O_{l}^{\prime }t$	Exported container by ship delayed	Containers	Variable	$O_{l}^{\prime }t=O_{l}t-\alpha$
	$Q_{m}^{\prime }$	Imported container by transportation mode m with delay	Containers	Variable	$Q_{m}^{\prime }=Q_{m}t-\beta ,\forall m=\left\{f,r,t\right\}$
	$V_{out}$	Volume of exported container in port	Containers	Variable	$V_{\mathrm{out}}=\int \left(O_{f}+O_{r}+O_{t}-O_{l}^{\prime }\right)dt$
	$V_{in}$	Volume of imported container in port	Containers	Variable	$V_{in}=\int \left(Q_{l}-Q_{f}^{\prime }-Q_{r}^{\prime }-Q_{t}^{\prime }\right)dt$
